# Evaluating the effectiveness and safety of ursodeoxycholic acid in treatment of intrahepatic cholestasis of pregnancy: A meta-analysis (a prisma-compliant study)

**DOI:** 10.1097/MD.0000000000004949

**Published:** 2016-10-07

**Authors:** Xiang Kong, Yan Kong, Fangyuan Zhang, Tingting Wang, Jin Yan

**Affiliations:** Department of Obstetrics and Gynecology, Clinical Medical College of Yangzhou University, Yangzhou, Jiangsu, China.

**Keywords:** intrahepatic cholestasis of pregnancy, meta-analysis, ursodeoxycholic acid

## Abstract

**Background:**

Intrahepatic cholestasis of pregnancy (ICP) is a specific pregnancy-related disorder without standard medical therapies. Ursodeoxycholic acid (UDCA) is the most used medicine, but the efficacy and safety of UDCA remain uncertain. Several meta-analyses had been made to assess the effects of UDCA in ICP. However, the samples were not large enough to convince obstetricians to use UDCA. We conducted a meta-analysis to evaluate the effects and safety of UDCA in patients with ICP, which included only randomized controlled trials (RCTs).

**Methods:**

Six databases were searched. The search terms were “ursodeoxycholicacid,” “therapy,” “management,” “treatment,” “intrahepatic cholestasis of pregnancy,” “obstetric cholestasis,” “recurrent jaundice of pregnancy,” “pruritus gravidarum,” “idiopathic jaundice of pregnancy,” “intrahepatic jaundice of pregnancy,” and “icterus gravidarum.”

Randomized controlled trials of UDCA versus control groups (included using other medicines) among patients with ICP were included. The primary outcomes were improved pruritus scores and liver function. Secondary outcomes were the maternal and fetal outcomes in patients with ICP.

Data were extracted from included RCTs. The Mantel–Haenzel random-effects model or fixed-effects model was used for meta-analysis.

**Results:**

A total of 12 RCTs involving 662 patients were included in the meta-analysis. In pooled analyses that compared UDCA with all controls, UDCA was associated with resolution of pruritus (risk ratio [RR], 1.68; 95% confidence interval [CI],1.12–2.52; *P* = 0.01),decrease of serum levels of alanine aminotransferase (ALT) (standardized mean difference (SMD), −1.36; 95% CI, −2.08 to −0.63; *P* <0.001), reduced serum levels of bile acid (SMD, −0.68; 95% CI, −1.15 to −0.20; *P* <0.001), fewer premature births (RR, 0.56; 95% CI, 0.43–0.72; *P* <0.001),reduced fetal distress (RR, 0.68; 95% CI, 0.49–0.94; *P* = 0.02), high Apgar scores at 5 minutes (RR, 0.44; 95% CI, 0.24–0.82; *P* = 0.009), less frequent respiratory distress syndrome (RDS) (RR, 0.33; 95% CI, 0.13–0.86; *P* = 0.02), and fewer neonates in the intensive care unit (NICU) (RR, 0.55; 95% CI, 0.35–0.87; *P* <0.05), increased gestational age (SMD,0.44; 95% CI, 0.26–0.63; *P* <0.001), and birth weight (SMD, 0.21; 95% CI, 0.02–0.40; *P* = 0.03). There were no differences in meconium staining and intrauterine growth retardation (IUGR) between the groups (*P* >0.05). No trials reported adverse effects on mothers and fetuses except nausea and emesis.

**Conclusion:**

UDCA is effective and safe to improve pruritus and liver function in ICP. UDCA also reduced adverse maternal and fetal outcomes in pregnant women with ICP.

## 1 Introduction

Intrahepatic cholestasis of pregnancy (ICP) is a unique pregnancy-related disorder, occurring during the late second or third trimesters of pregnancy. The clinical characters are unexplained maternal pruritus, altered liver functions (elevated serum transaminases), and increased fasting serum bile acids (>10 μmol/L) in previously healthy pregnant women.^[1,2]^ It is a reversible disease. After the strip of the placenta, signs and symptoms of ICP disappear.^[3]^ The incidence is variable geographically from 0.1% to15.6% all over the world.^[4,5]^ Currently, the etiology of this condition is not fully understood. Etiology seems to be multifactorial. Its pathogenesis is related to increased sex hormone synthesis, environmental factors, and genetic predisposition.^[6]^ The higher risk is to cause postpartum bleeding due to deficiency of vitamin K. Although ICP is a benign disease, ICP can lead to increased fetal morbidity and mortality, particularly with regards to preterm delivery, neonatal respiratory distress syndrome, fetal distress, and sudden intrauterine fetal death.^[7,8]^ ICP has no specific treatments until now. The treatments of the disease focus on relieving symptoms and signs because the pathophysiology is unclear. Cholestyramine, dexamethasone, S-Adenosyl-L-methionine (SAMe), and ursodeoxycholic acid have been used.^[9–12]^ Several studies had shown that ursodeoxycholic acid (UDCA) could improve itching and reduce the liver function tests in ICP.^[10,13]^ Gurung et al^[14]^ declared in the Cochrane collaboration that UDCA significantly improved itching as well as reduced the adverse fetal outcomes when compared with placebo but the difference was not statistically significant. Bacq et al^[15]^ evaluated 9 trials and found that UDCA was efficient to improve itching and ALT, what is more, it was beneficial to fetus. Grand’Maison et al^[16]^ evaluated 11 RCTs and 6 nonrandomized controlled trials (NRCTs), which suggested that UDCA could reduce adverse maternal and fetal outcomes. UDCA could stimulate the potassium channels to function as an antiarrhythmic and antifibrotic drug to prevent heart failure and fetal arrhythmia. UDCA can also decrease toxic endogenous bile acids by placental transfer of bile acids.^[17,18]^ However, there is still controversy about the real usefulness of this intervention. Optimal treatment mode of ICP is still controversial. The aims of the meta-analysis included RCTs were to evaluate the effects and safety of UDCA in the management of ICP.

## 2 Methods

We searched Medline, EMbase, PubMed, Web of science, the Cochrane Central Register of Controlled Trials, and Cochrane Library for articles published up to May 10, 2016. The search terms were “ursodeoxycholic acid,” “therapy,” “management,” “treatment,” “intrahepatic cholestasis of pregnancy,” “obstetric cholestasis,” “recurrent jaundice of pregnancy,” “pruritus gravidarum,” “idiopathic jaundice of pregnancy,” “intrahepatic jaundice of pregnancy,” “icterus gravidarum.” As meta-analysis does not involve patients, so the study does not require institutional ethics board approval. Studies were included if they met the criteria: the research population included patients diagnosed with ICP (itching, elevated bile acid, and liver function tests); the improvement of the clinical manifestations: relived itching, decreased liver enzymes; the outcomes of maternal and fetal: prematurity, birth weight, meconium staining, NICU, IUGR, and so on; RCTs. Studies were excluded if they reported as case series or observational studies and were not published in English. The primary outcomes were improved pruritus scores and liver function. Secondary outcomes were the maternal and fetal outcomes of ICP.

The titles and abstracts of all primary studies were screened by 2 researchers (XK and YK). Eligibility and potential risk of bias (including random sequence generation, allocation concealment, and blinding) were evaluated by 2 reviewers independently (XK and FZ). The third reviewer would be consulted if there was any disagreement between reviewers. We have tried to contact with the authors to obtain more details of their data including unpublished data so as to avoid heterogeneity and publication bias.

Data for basic information (name of the first author, year of publication, study style, number of participants), duration time and dose of UDCA, maternal and fetal outcomes including premature labor (gestational age <37 weeks), birth weight, number of caesarean sections, neonatal intensive care unit (NICU), and intrauterine growth restriction (IUGR, fetal weight below the 10th percentile for gestational age) were extracted from the included studies by 1 investigator (YK) and independently verified by another reviewer (TW). Any disagreement was resolved through discussion.

All results were merged for meta-analysis using Review Manager 5.3(The Nordic Cochrane Centre, Copenhagen, Denmark). The number of patients who were randomly assigned was regarded as the total number of participants in each study. Using the Mantel–Haenszel random-effects or fixed-effects model, outcomes were summarized through risk ratio (RR) or standardized mean difference (SMD) and 95% confidence intervals (CI) according to the styles of variable. We used RR and 95% CI to analyze dichotomous variable. Continuous variable were analyzed by SMD and 95% CI. Heterogeneity among the studies was assessed by the *χ*^2^ test and *I*^2^ (<25% deemed low heterogeneity, 25%–50% moderate; and >50% high) statistics. *P* <0.10 or *I*^2^>50% indicates that heterogeneity existed among the studies, so a random-effects model (Mantel–Haenszel method) must be used. Sensitivity analysis was assessed by using the leave-1-out approach.

## 3 Results

### 3.1 Identified studies

**Figure 1:**
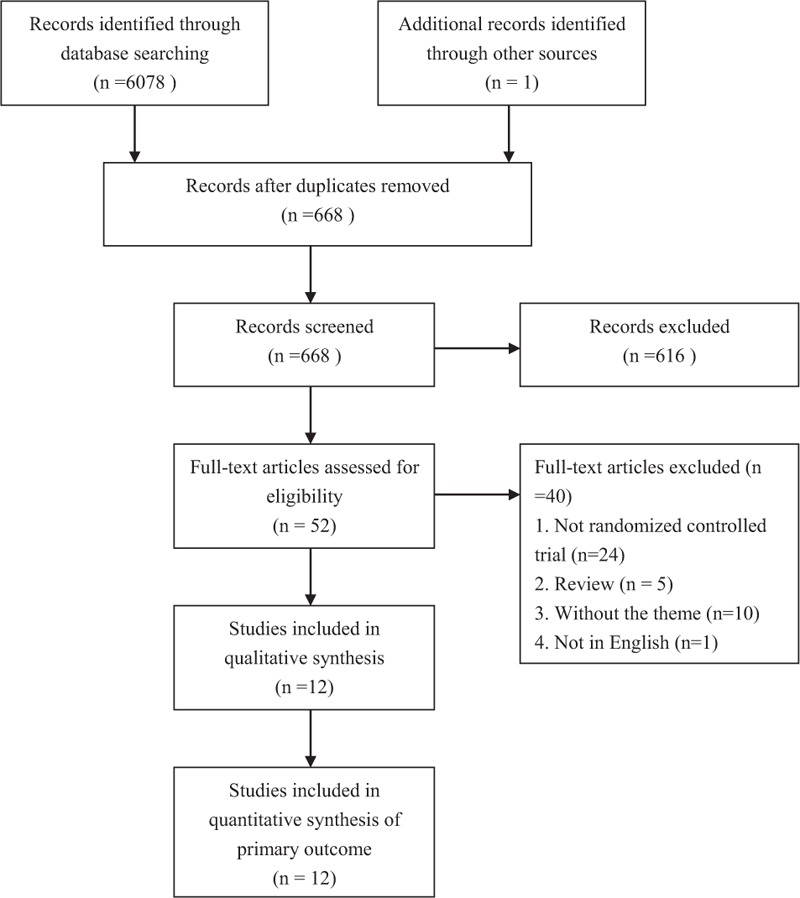
Flowchart of study selection.

**Figure 2:**
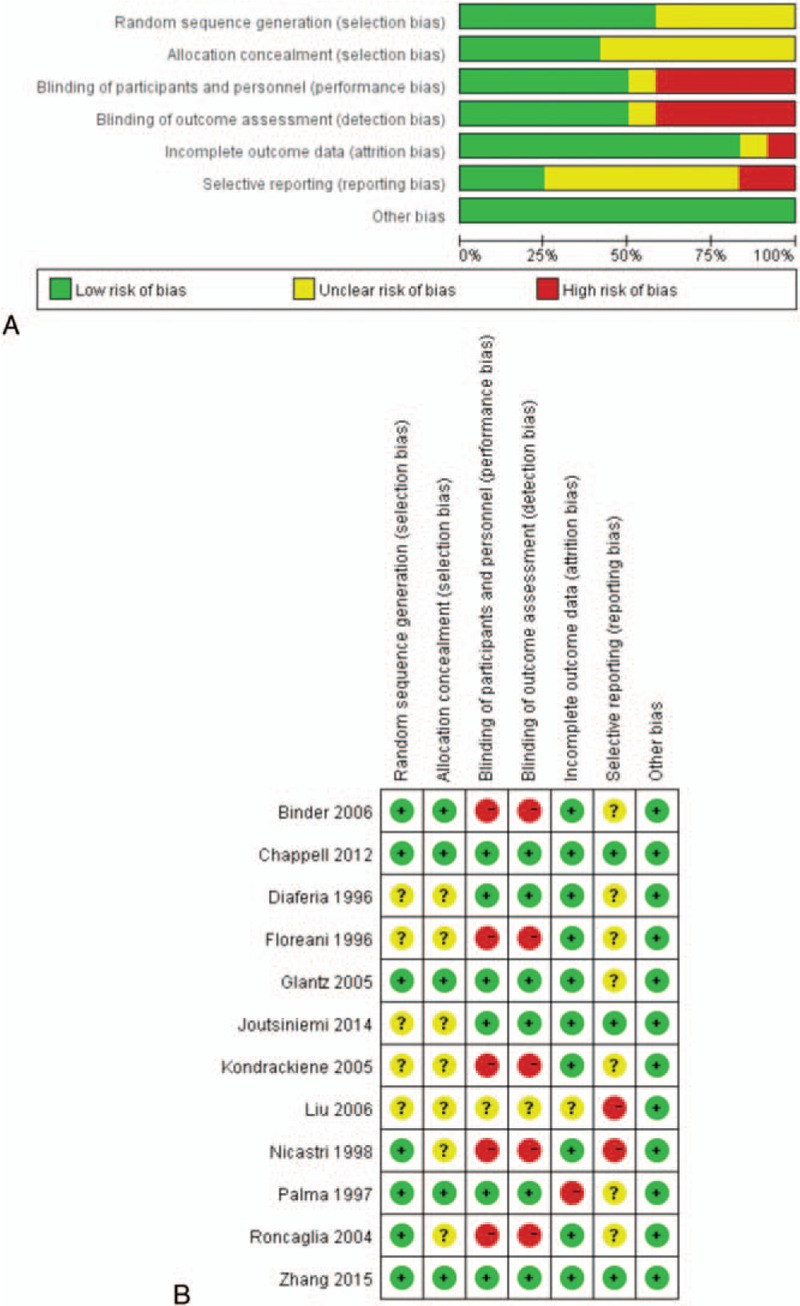
“Risk of bias” graph: review authors’ judgements about each risk of bias item presented as percentages across all included studies (A), “risk of bias” summary: review authors’ judgements about each risk of bias item for each included study (B).

After being assessed for eligibility, a total of 12 articles were included in the meta-analysis (Fig. 1). The results of the quality assessment of included studies are shown in Fig. 2A and B. The method of randomization was not clear in 3 trials.^[13,19,20]^ Some of the included studies did not state whether the analysis was intent-to-treat. Six researches did not use double blinding.^[12,19,21–24]^

The data extracted from the included studies are gathered in Table 1. The diagnosis of ICP was based on unexplained maternal pruritus and increased liver function (alanine aminotransferase (ALT), aspartate transaminase (AST), or total bile acid). UDCA was used to compare with several other drugs such as SAMe, placebo, dexamethasone. UDCA was also compared with the combination therapy of UDCA and SAMe in 3 studies.^[22,24,25]^ The subgroups (combination therapy of UDCA and SAMe) were excluded from the meta-analysis, which may cause publication bias. The present review only contained that UDCA compared with SAMe, placebo, or the other monotherapy.

**Table 1:**
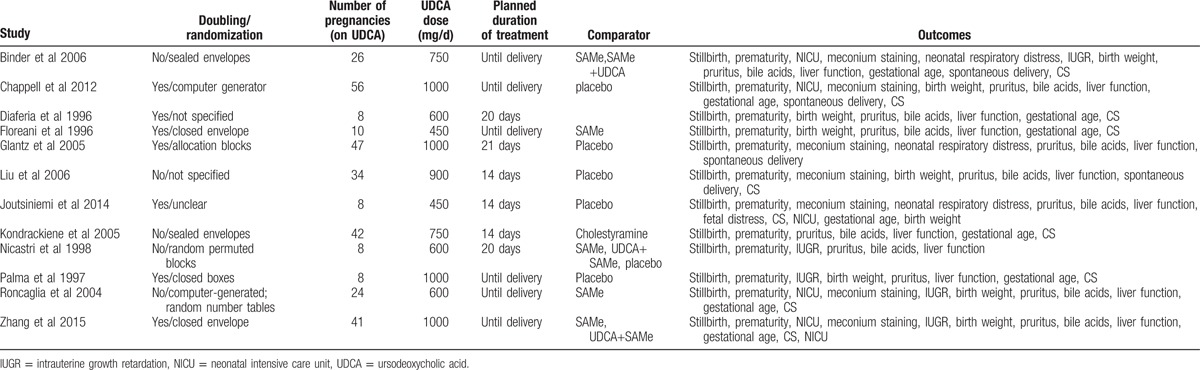
Characteristics of the studies included in the review.

### 3.2 Maternal outcomes

**Figure 3:**
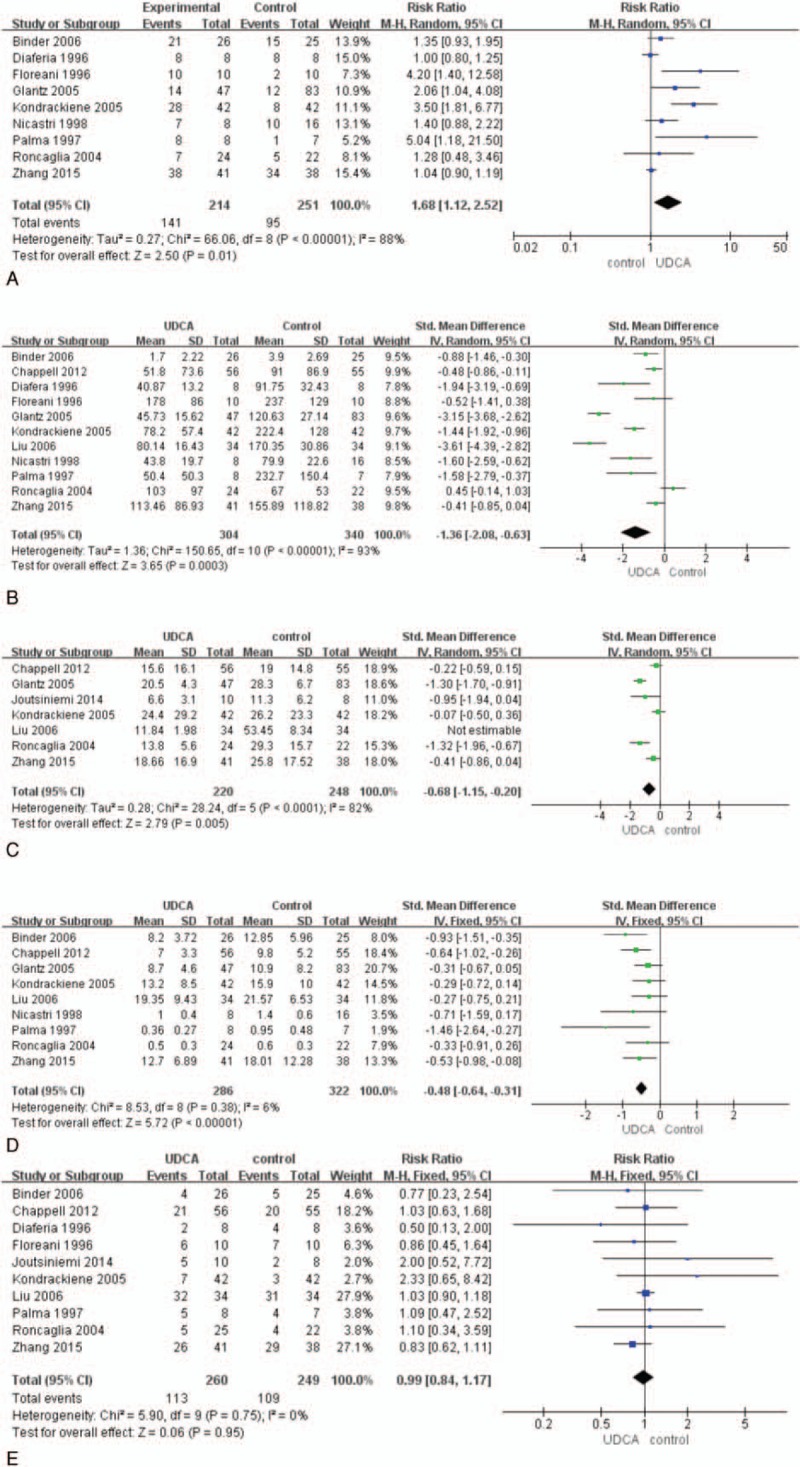
Forest plots of risk ratio for improvement of pruritus (A), serum ALT (B), bile acid (C), bilirubin (D), and caesarean section (E).

Of 12 studies (662 patients), 9 articles reported the improvement of maternal pruritus and were included in the meta-analysis (Fig. 3A). Visual analogue scales (10 cm) were used to assess the severity of maternal pruritus in 3 researches^[20,24,26]^ and categorical scales were used in 6 studies.^[10,13,21,22,25,27]^ The study^[21]^ that adopted the scales from 0 to 3 was different from the other categorical scales as they used 5 point scales. Five-point scales were used in 5 studies similarly:^[10,13,22,25,27]^ 0 = absence of pruritus, 1 = pruritus by accident, 2 = discontinuous pruritus every day, 3 = discontinuous pruritus but prevailing symptomatic lapses every day, 4 = pruritus all day and night. The 4-point scales used in 1 study were: 0 = absence of pruritus, 1 = occasional pruritus, 2 = discontinuous pruritus every day with prevailing symptomatic relapses at night, 3 = permanent pruritus during day and night.^[21]^ As the measurements of the pruritus were not coincident, the meta-analysis gave up analyzing continuous variable. The pooled analysis used the random-effects model as the *I*^2^ was 88% (high heterogeneity) and demonstrated significant difference in improving maternal pruritus by UDCA compared with control groups (RR 1.68, 95% CI 1.12–2.52; *P* = 0.01).

Eleven studies were included to assess UDCA in decreasing serum ALT compared with controls.^[10,12,13,19,21–27]^ The only 1 study was excluded from this analysis because the baseline of serum ALT levels in the groups of UDCA and control were significantly different at entry.^[20]^ As the measuring unit was not the same in the 11 included studies, we used SMD to assess the studies. The meta-analysis showed UDCA was more beneficial to improve maternal serum ALT levels. The difference was significant between UDCA and control groups (SMD −1.36, 95% CI −2.08 to −0.63; *P* <0.001). There was high heterogeneity between studies (*I*^2^ = 93%) (Fig. 3B).

We got the data of bile acid from 6 trials. But 1 of them was excluded because of the difference of the baseline. There was significant decrease in bile acid (SMD −0.68, 95% CI −1.15 to −0.20; *P* <0.001). Heterogeneity existed (*I*^2^ = 82%) (Fig. 3C).

Nine trials were included to analysis bilirubin and Diaferia study was excluded through sensitivity analysis. There was moderate heterogeneity between the trials (*I*^2^ = 6%). The improvement of bilirubin was significantly different (SMD −0.48, 95% CI −0.64 to −0.31; *P* <0.001) (Fig. 3D).

The results showed that 43% (113/260) patients adopted caesarean section in UDCA group, while 44% (109/249) patients occurred in control group. There were no difference in 2 therapies (RR 0.99, 95% CI 0.84–1.17; *P* = 0.95). There was no heterogeneity (*I*^2^ = 0%) (Fig. 3E).

### 3.3 Fetal and neonatal outcomes

Eleven RCTs had supplied the data of prematurity. Twenty-one percent (62/296) and 37% (123/332) pregnancies occurred premature labor in the compared groups, respectively. The pooled analysis showed that the rate of prematurity was lower in the UDCA groups than in control groups (RR 0.56; 95% CI 0.43–0.72; *P* <0.001). Heterogeneity did not exist (*I*^2^ = 0%) (Fig. 4 A).

**Figure 4:**
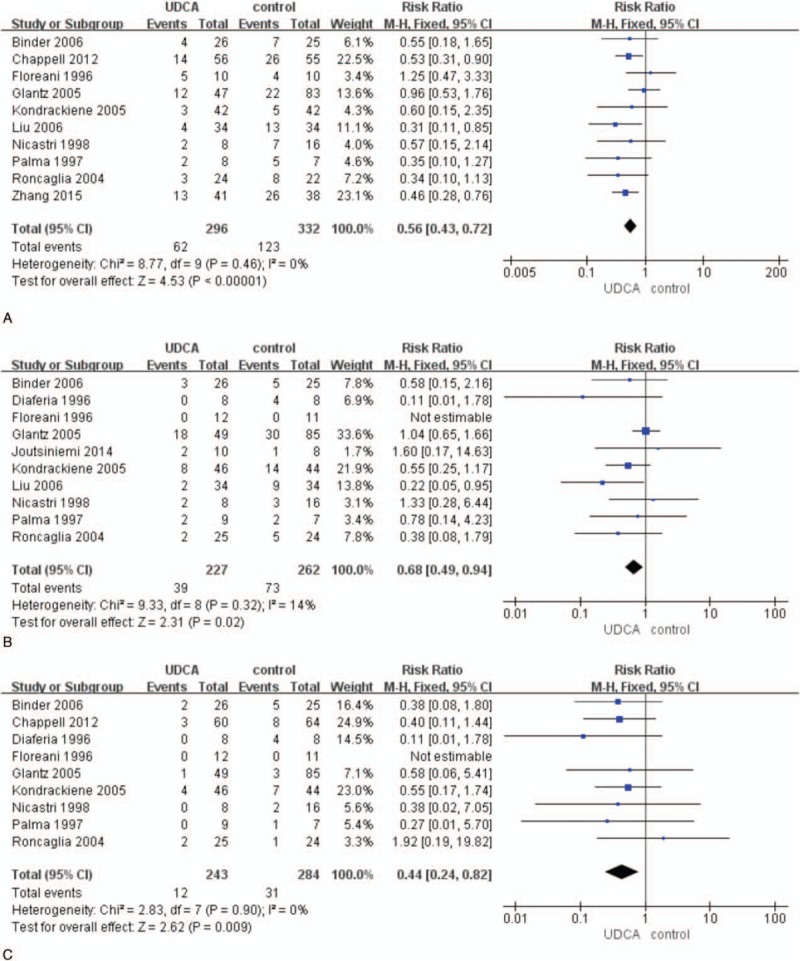
Forest plots of risk ratio for prematurity (A), fetal distress (B), Apgar scores <5 minutes (C), neonatal respiratory distress (D), NICU (E), gestational age (F), and birth weight (G).

**Figure 4 (Continued):**
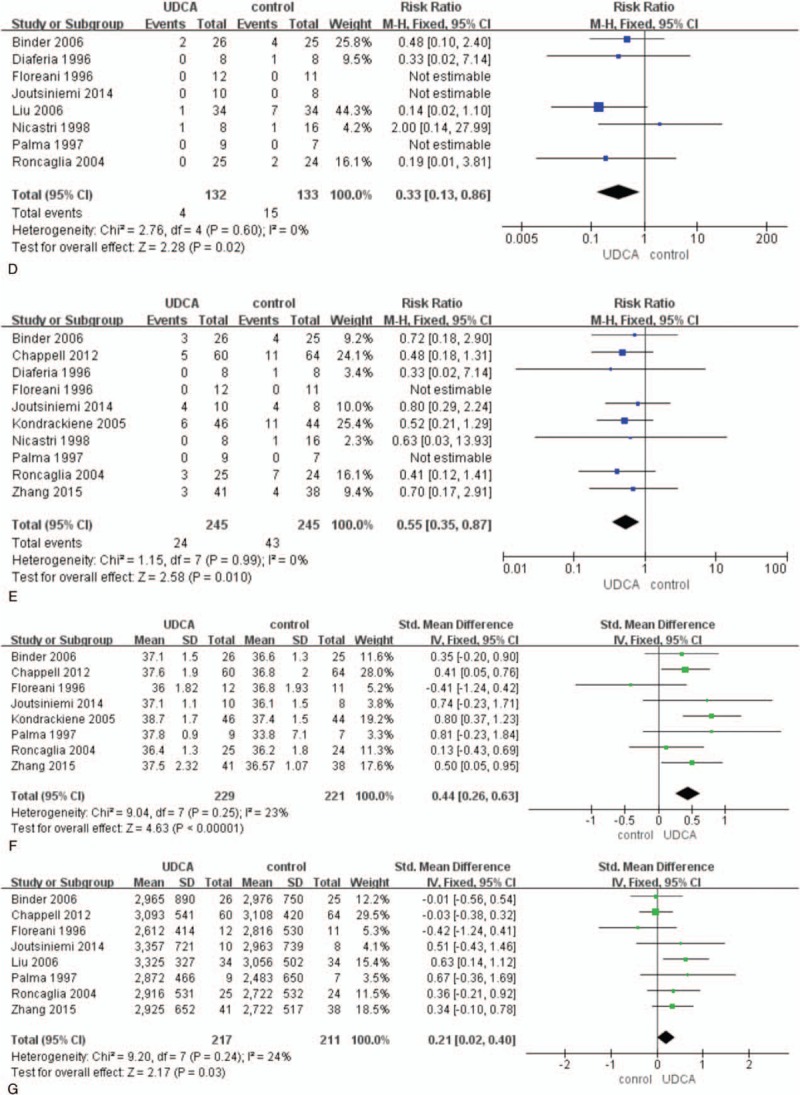
Forest plots of risk ratio for prematurity (A), fetal distress (B), Apgar scores <5 minutes (C), neonatal respiratory distress (D), NICU (E), gestational age (F), and birth weight (G).

The rate of IUGR was low and there was no significant difference in the groups (RR 1.22; 95% CI 0.43–3.48; *P* = 0.71). There was no difference in the rate of meconium staining in the meta-analysis. As meconium staining is not a sign for obstetricians to predict fetal distress, the analysis may have no significance. The fetal distress may be monitored by nonstress test. Thirty-nine of 227(17%) patients appeared fetal distress in the treatment group of UDCA and 73 of 262 (28%) patients had fetal distress in control groups. The difference was statistic significantly (RR 0.68; 95% CI 0.49–0.94; *P* = 0.02). Heterogeneity was small (*I*^2^ = 14%) (Fig. 4 B).

The rate of Apgar score <7 at 5 minutes was 4.9% (12/243), 10.9% (31/284) for UDCA and control groups, respectively. There was significant difference in the groups (RR 0.44; 95% CI 0.24–0.82; *P* = 0.009). Heterogeneity did not exist (*I*^2^ = 0%) (Fig. 4 C).

The rate of RDS was 3% (4/132), 11% (15/133) for UDCA and control groups, respectively. The difference was significant between the groups (RR 0.33; 95% CI 0.13–0.86; *P* = 0.02). Heterogeneity did not exist (*I*^2^ = 0%) (Fig. 4 D).

Twenty-four of 245(10%) newborns in UDCA group were admitted to NICU, which is less than 43 of 245(18%) in the control groups. The pooled analysis showed significant difference between the groups (RR 0.55; 95% CI 0.35–0.87; *P* = 0.01). There had no heterogeneity (*I*^2^ = 0%) (Fig. 4 E).

The gestational age in the treatment of UDCA group was longer than in the control groups and the difference was significant (SMD 0.44, 95% CI 0.26–0.63; *P* <0.001). Heterogeneity was moderate (*I*^2^ = 23%) (Fig. 4 F).

The birth weight in the treatment of UDCA group was higher than in the control groups and the difference was significant (SMD 0.21, 95% CI 0.02–0.40; *P* = 0.03). Heterogeneity was moderate (*I*^2^ = 24%) (Fig. 4 G).

### 3.4 Heterogeneity

Heterogeneity was significant for the outcomes of bilirubin, birth weight, and gestational age. The trial of Diaferia et al^[13]^ was mainly responsible for the heterogeneity. The study was excluded from the meta-analysis as the results of bilirubin, birth weight, and gestational age were different and the publication bias existed.

## 4 Discussion

UDCA is the most efficient drug to cure ICP, but there is no sufficient evidence to recommend UDCA alone or in combination with other drugs in treating women with ICP. Burrows et al^[28]^ reported that no evidence of positive effects for patients with ICP from taking UDCA. More researches were needed.

The present meta-analysis of 12 RCTs was conducted to assess the effects and safety of UDCA in ICP. The data showed a significant benefit for UDCA to improve maternal outcomes of pruritus and liver functions (ALT, bile acid, bilirubin). No significant differences existed in terms of caesarean section. UDCA was also found to significantly reduce the risk of fetal and neonatal adverse outcomes (excluded IUGR and meconium staining). These results support others’ findings.^[14–16]^ Our analysis also included 2 new articles written by Joustiniemi et al and Zhang et al.^[20,25]^ The study of Isla et al was also excluded from the meta-analysis as we could only read the abstract. The quality of the study was unclear.

We found high heterogeneity when analyzing improvement of pruritus, decrease of serum ALT and serum bile acid. This is likely due to the different ethnics, the different comparators, and different measurements of the outcomes.

In a previous meta-analysis, UDCA was effective in reducing pruritus and decreasing liver test results in patients with ICP.^[15]^ But Burrows et al^[28]^ reported UDCA was not proved to be significantly better than placebo in improving pruritus. We planned to dichotomise pruritus after test of intervention as “improvers” and “nonimprovers.” The result demonstrated a greater support for treatment of UDCA compared with controls in improving pruritus scores, which was coincident with the reports.^[14,15]^ Although ICP is a benign disease, it is necessary for obstetricians to use efficient drugs to relieve the very uncomfortable symptom as pruritus affects the live quality of the pregnant women.

A previous meta-analysis reported that the rate of neonatal respiratory distress was not decreased in women taking UDCA versus comparators^[14]^. But in the present meta-analysis, UDCA is benefit to decrease neonatal respiratory distress, which is different from Gurung's results.^[14]^ The Cochrane review reported there was no statistic differences in rate of fetal distress in the UDCA groups compared with placebo,^[14]^ but our results demonstrated that treatment of UDCA could reduce the risk of fetal distress. Bacq et al^[15]^ also concluded that UDCA might be beneficial to fetal outcomes as it reduced fetal distress and admissions to NICU. Some studies had reported that fetal and neonatal outcomes such as neonatal RDS and fetal distress were associated with maternal bile acid concentrations. Geenes et al^[29]^ found that serum bile acids ≥40 μmol/L was a sign to offer delivery from 37 weeks’ gestation and that may reduce the rate of stillbirths. Puljic et al^[30]^ also reported that induction of labor at 36 weeks’ gestation instead of expectant management could reduce the perinatal mortality risk. They thought the risk of stillbirth and the morbidity of preterm delivery may influence the time to delivery.^[30]^ It is evident that risk of adverse fetal outcomes is increased associated with maternal serum total bile acids elevated exceeding 40 umol/L.^[10,31]^ Currently, the mechanisms of the treatment of UDCA in cholestatic liver diseases are not completely revealed. The possible mechanisms are as follows: restore the normal metabolism of the liver; strengthen the bile acid transport across the placenta from the fetus to decrease bile acid accumulation; protect hepatic cell from toxicity of bile acids; increase the surface area of transport organization (e.g., terminal villi, vasodilator); prevent oxidative stress and apoptosis.^[2,32–38]^ The meta-analysis concluded that patients with ICP taking UDCA could decrease maternal bile acid concentrations and improve fetal outcomes.^[15,16]^

Two trials reported 2 fetal deaths in the control groups.^[10,27]^ The causes of the deaths were unclear. Two studies disclosed maternal adverse effects such as diarrhea and nausea, which may be due to the different populations and therapeutic dose. The instances of these side effects were few.^[10,27]^ So UDCA is well tolerated by pregnant women and adverse effects in newborns have not been observed.

The included studies of this meta-analysis existed several limitations. First, follow-up time was short, so the potential adverse effects would not be found. Only 2 studies reported that patients with ICP taking UDCA had nausea and dizziness. The causes of the adverse effects were unclear, which may be due to the different population and therapeutic dose. The symptom was not severe. According to these, we thought UDCA is safe, but the evidence is insufficient. More RCTs containing longer follow-up time will be welcome. Second, several studies did not describe the random methods and allocation procedures, which may result in bias of selection or performance. Third, we failed to demonstrate UDCA is effective on preventing intrauterine death, which is the most severe and feared consequence of ICP. In this meta-analysis, intrauterine death was too rare to allow conducting any analysis. It occurred in control groups.

The review supports the treatment of UDCA in patients with ICP as UDCA can improve maternal pruritus and decrease liver function tests. What is more, using UDCA is beneficial for fetal and neonatal outcomes. UDCA may reduce the trends of premature labor, admissions of NICU, fetal distress, and neonatal respiratory distress syndrome. The results may guide obstetricians to manage ICP. UDCA is effective and safe in the treatment of patients with ICP.
