# Evaluating the effectiveness and safety of ursodeoxycholic acid in treatment of intrahepatic cholestasis of pregnancy: A meta-analysis (a prisma-compliant study): Erratum

**DOI:** 10.1097/MD.0000000000006031

**Published:** 2017-01-20

In the article “Evaluating the effectiveness and safety of ursodeoxycholic acid in treatment of intrahepatic cholestasis of pregnancy: A meta-analysis (a prisma-compliant study)”,^[1]^ which appeared in Volume 95, Issue 40 of *Medicine*, Figure 1a originally appeared incorrectly. The figure should have appeared as follows:

**Figure:**
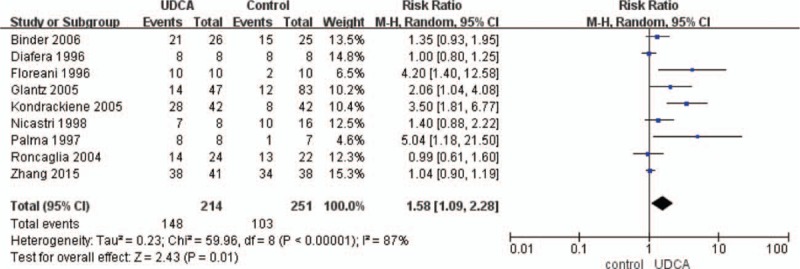


In addition, the final sentence of the first paragraph of section 3.2, Maternal Outcomes, should have read as follows:

The pooled analysis used the random-effects model as the *I*^2^ was 87% (high-heterogeneity) and demonstrated significant difference in improving maternal pruritis by UDCA compared with control groups (RR 1.58, 95% CI 1.09—2.28, *P* < 0.05).

## References

[1] Kong X Kong Y Zhang F Evaluating the effectiveness and safety of ursodeoxycholic acid in treatment of intrahepatic cholestasis of pregnancy: A meta-analysis (a prisma-compliant study). *Medicine.* 2016 95:e4949. 27749550 10.1097/MD.0000000000004949 PMC5059052

